# Compartment Syndrome following Open Femoral Fracture with an Isolated Femoral Vein Injury Treated with Acute Repair

**DOI:** 10.1155/2014/502657

**Published:** 2014-12-22

**Authors:** David Walmsley, Terry Axelrod, Sebastian Rodriguez-Elizalde

**Affiliations:** ^1^Division of Orthopaedic Surgery, Department of Surgery, University of Toronto, Room 508-A, 149 College Street, Toronto, ON, Canada M5T 1P5; ^2^Sunnybrook Health Sciences Centre, University of Toronto, Room MG-371, 2075 Bayview Avenue, Toronto, ON, Canada M4N 3M5; ^3^Humber River Regional Hospital, 810 Wilson Avenue, Toronto, ON, Canada M3K 1E5

## Abstract

Acute compartment syndrome is a surgical emergency and its diagnosis is more difficult in obtunded or insensate patients. We present the case of a 34-year-old woman who sustained a Gustilo-Anderson grade III open midshaft femur fracture with an isolated femoral vein injury treated with direct repair. She developed lower leg compartment syndrome at 48 hours postoperatively, necessitating fasciotomies. She was subsequently found to have a DVT in her femoral vein at the level of the repair and was started on therapeutic anticoagulation. This case highlights the importance of recognition of isolated venous injuries in a trauma setting as a risk factor for developing compartment syndrome.

## 1. Introduction

Acute compartment syndrome of the lower extremity is a potentially devastating complication that is most commonly associated with tibial diaphyseal fractures [1]. It requires a high index of suspicion particularly in obtunded or insensate patients. There are more rarely described etiologies of this condition, including massive fluid resuscitation in the absence of direct trauma [2], post-vein catheterization [3], post-deep-vein harvesting [4], and post-deep-vein thrombosis (DVT) [5, 6]. Early diagnosis and treatment with fasciotomies lead to improved outcomes [7, 8]. To our knowledge, there has not been a reported case in the literature describing acute lower leg compartment syndrome in the setting of acute venous injury and repair.

## 2. Case Presentation

**Figure 1:**
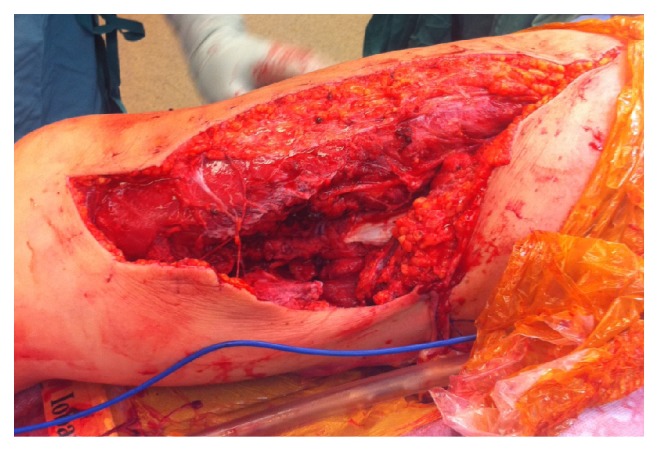
Intraoperative photograph of medial thigh wound after irrigation and debridement.

**Figure 2:**
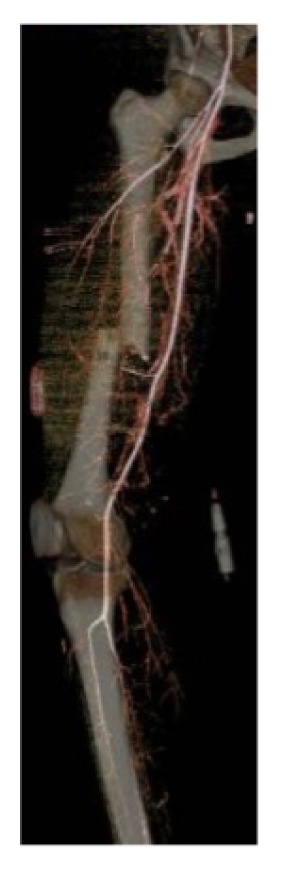
3D reconstruction of CTA angiogram performed preoperatively. No arterial injury was identified. Note the transverse middle third femoral shaft fracture.

A healthy 34-year-old woman operating a riding lawnmower lost control and collided with a tree. She was transferred directly to our level I trauma centre. She was hypotensive and tachycardic (sBP < 90 mmHg, HR > 120 bpm) on presentation, necessitating resuscitation with crystalloid and blood products. Her injuries included an open right femur fracture with a massive wound (Figure 1), an open left iliac wing fracture, and a closed left segmental humeral shaft fracture with associated radial nerve palsy, transverse process fractures of L3-5, a kidney laceration, and a bladder hematoma. Her Injury Severity Score was 41. On examination, there was no obvious injury to her right lower leg or foot. She had decreased sensation to pin prick and light touch globally in her right foot and calf with palpable dorsalis pedis (DP) and posterior tibial (PT) pulses. She had normal ankle dorsi and plantar flexion (5/5 MRC) with reduced toe flexion and extension (3/5 MRC). A tourniquet was immediately applied to her right upper thigh for approximately 30 minutes prior to a CT angiogram, which excluded an arterial injury (Figure 2). This was then reinflated for approximately 30 minutes prior to surgery.

**Figure 3:**
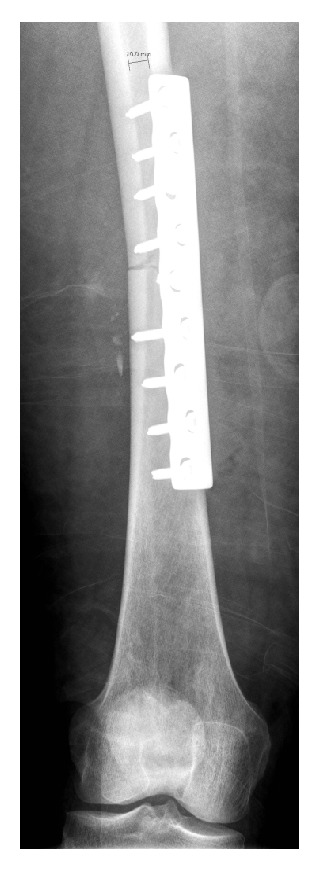
AP radiograph after temporary medial 4.5 mm LCP plate.

She was brought to the operating room within three hours of her injury and underwent irrigation and debridement of her right thigh and left iliac crest as well as temporary fixation of her femur fracture with a 4.5 mm compression plate (Figure 3). It was felt that this would provide the fastest stabilization of her femur fracture given her hemodynamic instability. Also, she would require repeat irrigation and debridement of her open wound at which point her fixation could be revised. She continued to bleed significantly and, after thorough exploration, a femoral vein laceration was identified. Vascular surgery repaired the vein with 5-0 interrupted Prolene sutures. Several tributaries to the femoral vein were ligated. The femoral artery was confirmed to be intact with a Doppler ultrasound above and below the level of the injury. The open wounds were loosely closed with interrupted nonabsorbable sutures. The estimated blood loss was 4 L intraoperatively. She received 12 units of packed red blood cells and eight units of fresh frozen plasma as well as 4 L of crystalloid and 3 L of colloid. She did not require vasopressors.

She was taken to the ICU in stable condition where she underwent further resuscitation. Her neurologic examination of her right leg remained unchanged immediately postoperatively. At 48 hours postoperatively, she developed significant calf swelling, without increased pain. Her compartments were firm and pulses were not palpable; however, Doppler signals were present for both DP and PT. She had weak plantar and dorsi flexion of her ankle (2/5 MRC), without any appreciable movement in her toes (0/5 MRC). Passive calf and toe stretching did not elicit pain. Compartment pressures were measured with a needle and revealed 49, 40, 36, and 33 mmHg in the anterior, deep posterior, superficial posterior, and lateral compartments, respectively. Her blood pressure at the time was 110/55 (MAP 73 mmHg). There was no concern about compartment syndrome in her thigh. She was brought urgently to the operating room within one hour of diagnosis to perform fasciotomies of her lower leg via a two-incision technique, with one incision medially to release the superficial and deep posterior compartments and one laterally to release the anterior and lateral compartments. There was immediate muscle bulging after fascial releases. All of her musculature appeared viable with good colour, consistency, bleeding, and contractility when stimulated by electrocautery. A VAC dressing was applied to both incisions. After fasciotomy, the patient had persistent decreased ankle dorsi and plantar flexion (2/5 MRC) and no toe extension or flexion (0/5 MRC). She had persistent global sensory loss in the calf and foot.

**Figure 4:**
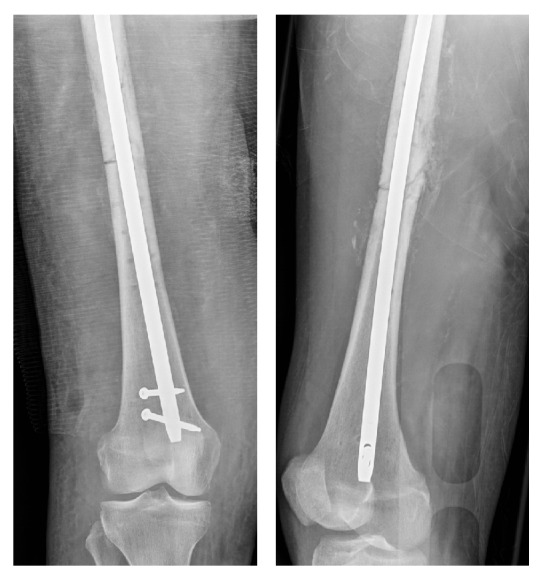
AP and lateral radiographs after revision fixation with an intramedullary nail.

After fasciotomy, a Doppler ultrasound demonstrated an occlusive DVT in her right femoral vein at the level of the venous repair. She was started on heparin until an IVC filter was inserted. At 72 hours after fasciotomy, she had conversion of the temporary medial femoral plate to a locked intramedullary nail (Figure 4), as well as repeat irrigation and debridement of her traumatic wound, and split-thickness skin grafts (STSG) for the traumatic wound and fasciotomy incisions. The lower leg fasciotomy sites were clean and the muscles were viable. She subsequently underwent definitive fixation of her left iliac wing fracture and left humerus fracture two weeks after her admission. She was restarted on heparin and the IVC filter was removed. She was then bridged to warfarin therapy.

Her course in hospital was complicated by a superficial infection of the STSG to her upper thigh, which resolved with IV antibiotics. She had a prolonged course of physiotherapy in a rehabilitation facility prior to discharge home. At 16 months after injury, she had sparse sensation throughout her foot and ankle. She had weak ankle plantar and dorsi flexion (3/5 MRC) and minimal toe flexion and extension (2/5 MRC). Her wounds and fractures were well healed. At two years after injury, she underwent revision of her fasciotomy scars using advancement flaps by plastic surgery. Given the severity of her initial injuries, the patient and her treatment team were satisfied with the final outcome.

## 3. Discussion 

Compartment syndrome in the setting of isolated arterial injuries and combined arterial and venous injuries is well described; however, its existence in the setting of acute, isolated venous injuries associated with trauma has not been reported. Traumatic venous injuries have been demonstrated in the following settings: low velocity gunshot wounds, stab wounds, blunt trauma, and shotgun wounds [9]. It is estimated that traumatic venous injuries occur without a concomitant arterial injury 25% of the time [7] and have been shown to occur 5–10% of the time in the popliteal vein [10]. Compromised venous outflow of the lower extremity has been implicated as causing compartment syndrome in 18% of patients undergoing deep vein harvesting for use in bypass procedures [4] as well as in a patient who developed thigh compartment syndrome after femoral vein catheterization [3].

The current treatment recommendation is for repair of venous injuries whenever it is technically possible and safe for the patient and for ligation otherwise [9]. Repair techniques vary based on the nature of the venous injury but lateral suture repair (lateral venorrhaphy) has yielded more successful outcomes than other techniques and is recommended whenever possible [9]. The benefits of repair are reduced incidence of postoperative venous hypertension and chronic venous insufficiency, improved arterial flow and patency, and improved limb salvage [15].

Controversy exists over the percentage of venous repairs that maintain patent over time; however, even short-term patency may allow for the establishment of venous and lymphatic collaterals and may lower the risk of developing compartment syndrome [9]. Of note, one study demonstrated that thrombosis occurred in 15% of patients undergoing popliteal vein repair and the authors recommended routine prophylaxis with higher doses of heparin and the use of IVC filers [11]. Deep venous thrombosis (DVT) has been shown to increase intracompartmental pressure in the lower extremity [12, 13]. Two cases have been reported of acute compartment syndromes occurring as a result of lower extremity DVT in the absence of other risk factors [5, 14]. Our patient occluded her femoral vein at the repair site, which in the setting of impaired collateral flow from her soft tissue injury likely led to the development of compartment syndrome.

Prophylactic fasciotomies are recommended in the setting of combined arterial and venous injuries when restoration of circulation is delayed, when venous repair is not adequate or the vein requires ligation, or when injuries to the thigh impede venous collateral outflow [15, 16]. Fasciotomies of the lower extremity are not benign procedures and complications include chronic pain, infection, nerve injury, disfiguring wounds particularly when they require skin grafts, and chronic venous insufficiency [16]. However, the functional outcomes are improved when they are performed within 12 hours of compartment syndrome onset [8].

In closing, this case emphasizes the importance of recognizing isolated venous injuries associated with trauma as a risk factor for acute compartment syndrome.

## Appendix

See Figures 1, 2, 3, and 4.
